# Genomic evidence for putative insertion sequence-associated chromosomal integration of aerobactin virulence loci in ST11 carbapenem-resistant *Klebsiella pneumoniae*

**DOI:** 10.3389/fmicb.2026.1855380

**Published:** 2026-09-10

**Authors:** Na Wang, Xiaocui Peng, Huiying Li, Xiaoyu Li, Chunmei Lei, Bu Wang, Jianhua Liu, Wei Zhang

**Affiliations:** 1 Microbiology Department, The First Affiliated Hospital of Hebei North University, Zhangjiakou, Hebei, China; 2 Infection Management Department, The First Affiliated Hospital of Hebei North University, Zhangjiakou, Hebei, China; 3 Hebei Key Laboratory of Pathogenic Mechanisms and Diagnosis & Treatment Technologies for Lung Microbiome, The First Affiliated Hospital of Hebei North University, Zhangjiakou, Hebei, China; 4 Respiratory Department, The First Affiliated Hospital of Hebei North University, Zhangjiakou, Hebei, China; 5 Obstetrics and Gynecology Department, The First Affiliated Hospital of Hebei North University, Zhangjiakou, Hebei, China; 6 Central Laboratory, The First Affiliated Hospital of Hebei North University, Zhangjiakou, Hebei, China

**Keywords:** candidate chromosome-associated virulence loci, genomic surveillance, hypervirulent carbapenem-resistant *Klebsiella pneumoniae*, ST11-KL64, virulence loci

## Abstract

**Background:**

The global rise in hypervirulent carbapenem-resistant *Klebsiella pneumoniae* (hv-CRKP) poses a severe threat to public health. However, the evolutionary mechanisms underlying hypervirulence in carbapenem-resistant strains remain incompletely understood. Here, we investigated the genetic basis of hypervirulence in a prevalent carbapenem-resistant lineage.

**Methods:**

Whole-genome sequencing and comparative genomic analyses were performed on 112 locally collected carbapenem-resistant *Klebsiella pneumoniae* (CRKP) clinical isolates collected from 2020 to 2022 and 4,636 publicly available *K. pneumoniae* genomes, including 557 hv-CRKP.

**Results:**

Among 66 hv-CRKP identified locally, the ST11-KL64 lineage was dominant, accounting for 87.9%. As against the classical fusion-plasmid model, these isolates harbored candidate chromosomal virulence-locus configurations, supported by convergent short-read sequencing evidence, with insertion sequences identified at or near the inferred breakpoint regions. IS-positive hv-CRKP isolates included in the phenotypic comparison showed significantly greater virulence in *Galleria mellonella* infection models than the IS-negative hv-CRKP comparator isolates (log-rank test, *p* < 0.0001). Global phylogenetic analysis showed that strains carrying related candidate chromosomal virulence-locus configurations clustered within a distinct lineage, with the currently available genomes in this group predominantly originating from China.

**Conclusion:**

This study identified recurrent candidate chromosome-associated virulence-locus configurations in ST11 hv-CRKP that are distinct from canonical fusion-plasmid architectures. This evidence extends the current models beyond plasmid fusion and underscores the value of genomic surveillance strategies capable of detecting chromosomal virulence, which may be overlooked when surveillance focuses solely on virulence plasmids. Defining this evolutionary trajectory provides a foundation for infection prevention and control, as well as for designing targeted interventions to limit the spread of these high-risk pathogens. Using multiple lines of genomic evidence, we established a framework for assessing candidate chromosomal localizations and identifying breakpoint-associated read signatures.

## 1 Introduction

*Klebsiella pneumoniae* is a major opportunistic pathogen responsible for several healthcare-associated infections, including pneumonia, bloodstream infections, urinary tract infections, and intra-abdominal infections (Pu et al., 2023; Shi et al., 2024). The global rise in carbapenem-resistant *K. pneumoniae* (CRKP) has created substantial challenges for clinical antimicrobial therapy (Zhou et al., 2021; Zhao et al., 2019; World Health Organization, 2017; World Health Organization, 2024). Notably, the convergence of carbapenem resistance with traits characteristic of hypervirulent *K. pneumoniae* (hvKP) is of concern (Russo et al., 2018), particularly the hypermucoviscous phenotype resulting in the emergence of hypervirulent carbapenem-resistant *Klebsiella pneumoniae* (hv-CRKP) (Jiang et al., 2025b; Wei et al., 2025).

Conventional models suggest that hv-CRKP primarily emerges through acquisition of resistance plasmids by hvKP or acquisition of virulence plasmids by CRKP, with plasmid recombination occasionally generating resistance–virulence hybrid plasmids (Liu et al., 2020; Zhang, et al., 2020; Xie et al., 2021; Yang et al., 2021). Such evolutionary convergence is facilitated by the highly plastic accessory genome of *K. pneumoniae* and the mobility of resistance- and virulence-associated genetic elements (Wyres et al., 2020). The aerobactin (iuc) and salmochelin (iro) loci are commonly associated with virulence plasmids, but their genetic backgrounds and mobilization patterns are diverse (Lam et al., 2018; Tian et al., 2021). Although hypervirulence-associated loci are predominantly plasmid-borne, rare chromosomal integration events involving the iuc operon have also been reported (Yang et al., 2020). However, the prevalence, genetic mechanisms, and epidemiological implications of chromosomal integration of virulence loci in major hv-CRKP clones remain incompletely understood.

The ST11 clonal complex is the predominant CRKP lineage in China, and ST11-KL64 hypervirulent carbapenem-resistant isolates have also been reported outside China, including the United States (Hu et al., 2024; Zhou et al., 2020; Jiang et al., 2025a). Our long-term surveillance revealed an atypical genetic feature in locally circulating ST11-KL64 hv-CRKP strains, wherein these strains consistently expressed hypervirulence, but lacked identifiable replicons of canonical hypervirulent plasmids. This finding suggests an alternative evolutionary mechanism for acquiring hypervirulence, independent of plasmid fusion events.

In this study, we combined longitudinal surveillance, comparative genomics of local and global isolates, and functional assays. These approaches allowed us to define the population structure and molecular features of regional hv-CRKP and to elucidate the genetic basis of hypervirulence in the ST11-KL64 lineage, with a focus on putative insertion sequence-associated chromosomal integration of virulence loci. Additionally, we assessed the biological and epidemiological relevance of this mechanism for clonal emergence and dissemination, as well as its implications for infection prevention and control.

## 2 Materials and methods

### 2.1 Bacterial strain collection and identification

A total of 112 non-redundant CRKP clinical isolates were retrospectively collected from the First Affiliated Hospital of Hebei North University (tertiary Grade A hospital) between 2020 and 2022. Among them, 76 isolates were part of a previously published collection from our group (Wang et al., 2025), and 36 isolates were newly included in the present study. Species identification was initially performed using a BD Phoenix 100 system (Becton, Dickinson and Company, NJ, USA) and confirmed using matrix-assisted laser desorption/ionization time-of-flight mass spectrometry (MALDI-TOF MS; Autobio, China). Antimicrobial susceptibility testing was conducted by broth microdilution, and MICs were interpreted according to CLSI M100 (ED33). Isolates with MICs ≥ 4 mg/L for imipenem or meropenem were defined as CRKP.

Among the CRKP isolates, 66 were classified as genetically defined hypervirulent carbapenem-resistant *Klebsiella pneumoniae* (hv-CRKP). In this study, hv-CRKP was operationally defined as a CRKP isolate carrying at least one hypervirulence-associated genetic marker, including *iucA*, *iroB*, *rmpA*, or *rmpA2*, as identified by whole-genome sequencing. This classification did not require the simultaneous presence of all hypervirulence-associated genes. This genotype-based classification does not necessarily indicate phenotypic confirmation of hypervirulence.

### 2.2 Public genome data acquisition and screening

For global comparison and phylogenetic analyses, genome assemblies and associated metadata for 4,636 fully sequenced *K. pneumoniae* isolates were retrieved from the NCBI Pathogens database. All genomes were processed in PathogenWatch for standardized quality control, molecular typing, and gene annotation, resulting in 3,637 high-quality genomes with complete annotations for downstream analyses (Argimón, et al., 2021). From these, 557 isolates co-harboring carbapenemase genes (e.g., *bla*KPC, *bla*NDM, *bla*OXA-48, *bla*IMP, and *bla*VIM) and hypervirulence-associated markers (e.g., *iucA, iroB,* and *rmpA/rmpA2*) were selected to investigate global prevalence patterns and evolutionary relationships. In addition, 100 publicly available isolates carrying classical fusion plasmids (supported by published reports and genomic annotation) were included as a comparator set for plasmid evolution analyses. All accession numbers and corresponding metadata for the strains and plasmids used in this study are provided in Supplementary Tables S1A–C.

### 2.3 Whole-genome sequencing and genome assembly

Genomic DNA was extracted using an SDS lysis method. DNA integrity was assessed by 1% agarose gel electrophoresis, and DNA concentration was quantified using a Qubit 2.0 fluorometer (Thermo Fisher Scientific, USA). Sequencing libraries were prepared using the NEBNext Ultra DNA Library Prep Kit for Illumina (New England Biolabs, Ipswich, MA, USA), following the manufacturer’s instructions. Briefly, 1 μg of genomic DNA per sample was fragmented to an average size of approximately 350 bp by ultrasonication, followed by end repair, A-tailing, adapter ligation, PCR amplification, and purification using AMPure XP beads (Beckman Coulter, USA). Library fragment size distribution was evaluated using an Agilent 2,100 Bioanalyzer, and library concentration was determined by qPCR. Paired-end sequencing (2 × 150 bp) was performed using the Illumina NovaSeq 6,000 platform. Genome assembly statistics for all isolates are provided in Supplementary Table S2.

Raw reads were filtered to obtain clean reads by removing reads with >40% bases having Q ≤ 20, reads containing >10% ambiguous bases (N), and reads with adapter overlap >15 bp, allowing ≤3 mismatches. *De novo* assemblies were generated using SOAPdenovo (k = 95, 107, 119), SPAdes (k = 99, 127), and ABySS (k = 64). For each isolate, the assembly with the fewest scaffolds and the largest N50 was retained. Optimal assemblies were merged using CISA to produce a consensus assembly, followed by gap filling with GapCloser. Scaffolds shorter than 500 bp were removed prior to downstream analyses.

### 2.4 Molecular typing, gene annotation, phylogenetics, and plasmid/MGE analyses

Sequence types (STs), capsule loci (K-locus; KL), and O-antigen loci (O-locus; OL) were predicted using PathogenWatch. Virulence determinants and virulence scores were obtained using the Kleborate-based module within PathogenWatch, and additional screening was performed using ABRicate (v1.0.1) against VFDB. Antimicrobial resistance genes were identified using ABRicate against the ResFinder database. ABRicate screening was performed using the default thresholds (minimum 80% nucleotide identity and 80% coverage). Gene calls were manually curated when discrepancies were observed between tools/databases.

Core-genome SNPs were identified using Snippy (v4.6.0) with *K. pneumoniae* TVGHCRE225 (GCA_003203435.1) as the reference genome. The Snippy-core SNP alignment (core.aln) was used for phylogenetic inference, whereas the full core-genome alignment (core.full.aln) was retained for reference. For the focused analysis of the 22 high-confidence integration candidates, the reference-excluded core-SNP alignment comprised 2,417 variable sites. A maximum-likelihood phylogenetic tree was constructed using FastTree (Price et al., 2010) and visualized using iTOL (Letunic and Bork, 2024), with tree files pre-processed in R for publication-quality rendering where required. Pairwise SNP distances among the 22 isolates were calculated from the reference-excluded core-SNP alignment using snp-dists v1.2.0.

Insertion sequences (ISs) were identified by alignment against the ISfinder database. Plasmid replicon types were predicted using MOB-suite (Robertson and Nash, 2018) and ABRicate with PlasmidFinder. Localization of virulence loci was first inferred using mlplasmids (Arredondo-Alonso et al., 2018) and then supported by manual BLAST-based contig/scaffold alignment. This workflow identified 23 ST11 isolates carrying candidate chromosome-associated virulence-locus configurations. These candidates were subjected to the multi-evidence assessment described below. Following this assessment, 22 isolates were retained as high-confidence integration candidates and were included in focused downstream phylogenetic and plasmid evolution analyses. To assess relatedness between resistance plasmids from these candidates and globally described fusion plasmids, Mash (Ondov et al., 2016) was used to compute pairwise distances among 22 assembled *bla*KPC-2-carrying resistance plasmids contigs and 100 reference fusion plasmids, followed by tree reconstruction based on the distance matrix and visualization in iTOL.

### 2.5 Multi-dimensional validation of chromosomal integration evidence and group assignment

For the 23 ST11 candidate isolates, candidate integration contigs were required to be ≥40 kb and contain complete virulence loci (*iucABCD-iutA* and *rmpA2*). To sensitively seed candidate chromosomal-marker detection in potentially divergent integration-associated regions, housekeeping gene homologs (*gyrB, rpoB, recA, and rplB*) were queried on the same contig by tBLASTn using *K. pneumoniae* protein queries obtained from NCBI. A relaxed E-value threshold of 10 was applied given divergence in putative chromosomally integrated regions, and hits were manually inspected for alignment coverage and expected functional characteristics. The relaxed tBLASTn threshold was used only for candidate seeding and was not considered sufficient on its own for integration assignment. The multi-evidence framework additionally included assessment of the corresponding pre-merge SPAdes assembly graph for all 23 candidate isolates, as described below. CISA-derived scaffold joins were not treated as standalone evidence of physical chromosome–virulence linkage. After applying the multi-evidence framework, 22 isolates met all criteria and were considered high-confidence integration candidates, whereas one isolate (Kpn_91) was excluded because manual BLAST-based curation provided insufficient support for its classification as a high-confidence integration candidate.

Putative integration patterns were categorized into four groups (Group A–D) based on differences in region size, gene order, and flanking sequences, together with housekeeping-gene similarity profiles. Associations between integration pattern groups and phylogenetic structure were evaluated using Fisher’s exact test. For detailed structural visualization and figure illustration, one representative isolate from each group was selected: Kpn_121 (Group A), Kpn_82 (Group B), Kpn_29 (Group C), and Kpn_63 (Group D).

### 2.6 Read-mapping visualization and breakpoint-support assessment of the putative integration region

Illumina reads were aligned to the *Klebsiella pneumoniae* ATCC 13883 reference assembly (GCF_000742135.1), specifically the NZ_KN046818.1 contig, using BWA-MEM with default parameters. Alignments were coordinate-sorted and indexed, PCR/optical duplicates were removed, and only reads with MAPQ ≥ 30 were retained for visualization and downstream analyses. Candidate putative integration breakpoints were inspected in IGV (v2.16.2) using ±200 bp windows centered on the breakpoint coordinates. The representative IGV snapshots shown in this study correspond to NZ_KN046818.1:1,817,330–1,817,730 (A breakpoint at 1,817,530) and NZ_KN046818.1:1,828,803–1,829,203 (B breakpoint at 1,829,003).

Meanwhile, we parsed SPAdes assembly graph summary outputs using custom Python scripts to generate a standardized set of graph-derived descriptors for candidate integration contigs across isolates. Candidate contigs were indexed by sample and SPAdes node identifier (e.g., NODE_x_length_y_cov_z); when multiple candidate nodes were present within an isolate, we retained a single representative node by selecting the longest contig. For each representative node, we summarized contig size and coverage together with assembly-graph topology features, including the oriented segment traversal (strand-annotated segment path), terminal segments, end-node degrees, and immediate neighbor sets, and used these descriptors to assess local contig coherence and flag potential assembly artifacts, particularly in repetitive regions.

Custom Python scripts were used for quantitative summaries of breakpoint-supporting signals at loci A and B, including the number of soft-clipped reads within ±5 bp of the breakpoint (clip ±5), the number of reads with supplementary alignments within ±50 bp (SA ± 50), and local depth calculated over a 500-bp window (DP500). Metrics were compared across isolates using log(1 + count), and evidence heatmaps were generated from per-metric z-scores. The genomic distance between A and B breakpoints was computed as |A_bp − B_bp|. These breakpoint-support metrics were used as supportive quantitative summaries rather than as standalone decision rules or direct proof of physical chromosome–virulence linkage.

A customized Python script quantified per-base read depth across the putative integration locus for all strains and calculated the proportion of zero-coverage sites. Summary visualizations included mean coverage per strain (with the overall mean and a 200 × reference threshold), per-strain coverage distribution box plots, zero-coverage percentage plots (1% quality threshold), and coverage heatmaps showing continuous depth variation along the locus. The script used pandas (v1.5.3), numpy (v1.24.3), matplotlib (v3.7.1), and seaborn (v0.12.2) libraries.

### 2.7 Reference-sensitivity analysis using a closer ST11 reference

To assess the potential effect of reference divergence on read mapping and breakpoint-associated signals, we compared mapping results obtained with ATCC 13883 and the closer ST11 reference TVGHCRE225. For the chromosome-level comparison, reads were aligned to ATCC 13883 and TVGHCRE225 chromosome CP023722.1 using the same mapping and filtering procedures. Summary statistics were calculated across all 22 high-confidence candidate isolates. Per-isolate mapping statistics for ATCC 13883 and TVGHCRE225 are provided in Supplementary Table S3A.

For the candidate breakpoint-associated regions, the complete TVGHCRE225 assembly was used, including plasmid contig CP023723.1. Homologous regions corresponding to the ATCC 13883 candidate coordinates were identified by BLAST-based comparison. We then examined soft-clipped and supplementary-alignment reads at the corresponding homologous regions. A complete junction-signature reproduction required concordant read-level evidence supporting both candidate junctions in the closer-reference analysis. Isolates with one or more soft-clipped or supplementary-alignment reads but without complete reproduction of the original junction signature were classified as showing weak residual signal. Isolates with mapping-compatible homologous regions but no qualifying read-level junction evidence were classified as mapping-compatible only. Per-isolate classification and breakpoint-associated read-level results from the TVGHCRE225 analysis are provided in Supplementary Table S3B.

### 2.8 Pre-merge SPAdes assembly-graph assessment of candidate integration contigs

To assess whether candidate integration contigs reflected coherent genomic paths rather than assembly artifacts, we performed assembly assisted graph assessment using pre-merge SPAdes assembly graphs. Draft genomes were assembled using SPAdes as described above, and assembly graphs were evaluated using Bandage to trace graph connectivity around candidate virulence locus-containing contigs. For each candidate integration contig, we recorded the corresponding SPAdes node identifier, neighboring graph nodes directly connected to the target node, and the ordered node path supporting the target contig within the assembly graph when a unique traversable path could be identified. These graph-based summaries were used as complementary evidence of local contig coherence and graph connectivity. They were not interpreted as direct validation of every junction potentially introduced during CISA merging or as standalone proof of physical chromosome–virulence linkage. Meanwhile, candidate contig segments were aligned to the *K. pneumoniae* ATCC 13883 reference assembly (GCF_000742135.1) using nucleotide alignment, and alignment identity and span (pident, aligned length, and reference coordinates) were used as supportive context. All assembly-graph, read-mapping, and contig-coverage summaries for the retained candidate isolates are provided in Supplementary Table S4.

### 2.9 Virulence phenotype assay

Healthy final-instar larvae (250–350 mg) were randomly assigned to treatment groups. The IS-positive hv-CRKP group included 15 distinct strains, comprising 7 IS-positive KL47 strains and 8 IS-positive KL64 strains, while the IS-negative hv-CRKP group included 10 distinct strains. Ten larvae were tested per strain per condition in each experiment. Each experiment was repeated in three independent biological replicates using independent larval batches prepared on different days. PBS-injected larvae served as the non-infected control. The larvae were incubated at 35 °C in the dark and monitored every 4 h for 24 h. Survival curves were compared using the log-rank test, and *p* < 0.05 was considered statistically significant.

### 2.10 Statistical analysis

All statistical analyses were performed in R (v4.2.1; R Foundation for Statistical Computing, Vienna, Austria). Categorical comparisons were performed using Fisher’s exact test where appropriate. Survival analyses for the *Galleria mellonella* model were conducted using the log-rank test in GraphPad Prism 8 (GraphPad Software, San Diego, CA, USA). A two-sided *p* value < 0.05 was considered statistically significant.

## 3 Results

### 3.1 Molecular epidemiology and genomic features of the dominant ST11-KL64 hv-CRKP

#### 3.1.1 Molecular characteristics of the dominant clone in the local cohort

**Figure 1:**
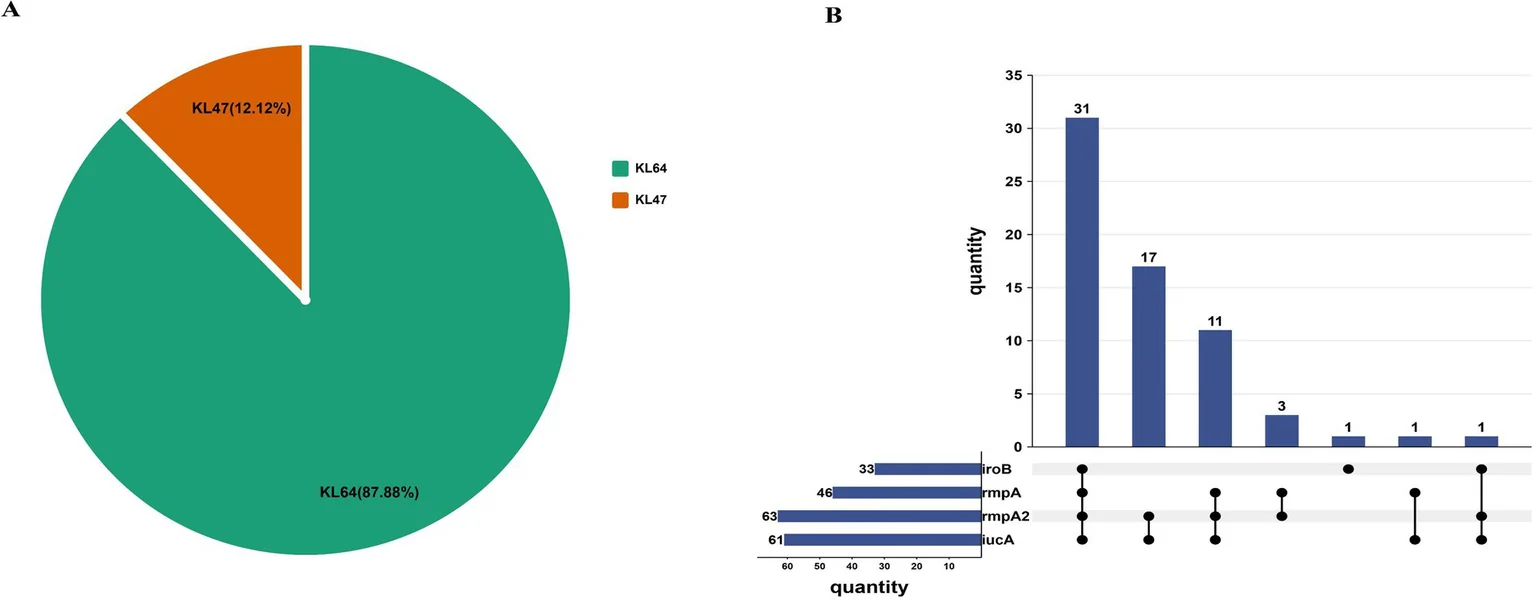
Molecular characteristics of the dominant hv-CRKP clone in this study. **(A)** Proportions and ST/KL distribution of local hv-CRKP. **(B)** UpSet plot of virulence gene combinations in hv-CRKP.

Among 112 locally collected CRKP isolates, 66 (58.9%) were classified as hv-CRKP. All hv-CRKP isolates belonged to ST11 (66/66, 100%), and KL64 predominated (58/66, 87.9%), establishing ST11-KL64 as the dominant local hv-CRKP lineage (Figure 1A). The clinical and molecular characteristics of the 112 *K. pneumoniae* isolates are summarized in Supplementary Table S5. Virulence profiling of the 66 hv-CRKP isolates showed frequent detection of *iucA* (61/66) and *rmpA2* (63/66), followed by *rmpA* (46/66) and *iroB* (33/66). At the genotype-combination level, the most prevalent profiles were *iroB/iucA/rmpA/rmpA2* (47.0%), *iucA/rmpA2* (25.8%), and *iucA/rmpA/rmpA2* (16.7%) (Figure 1B).

#### 3.1.2 Global prevalence and phylogenetic position of ST11-KL64 hv-CRKP

**Figure 2:**
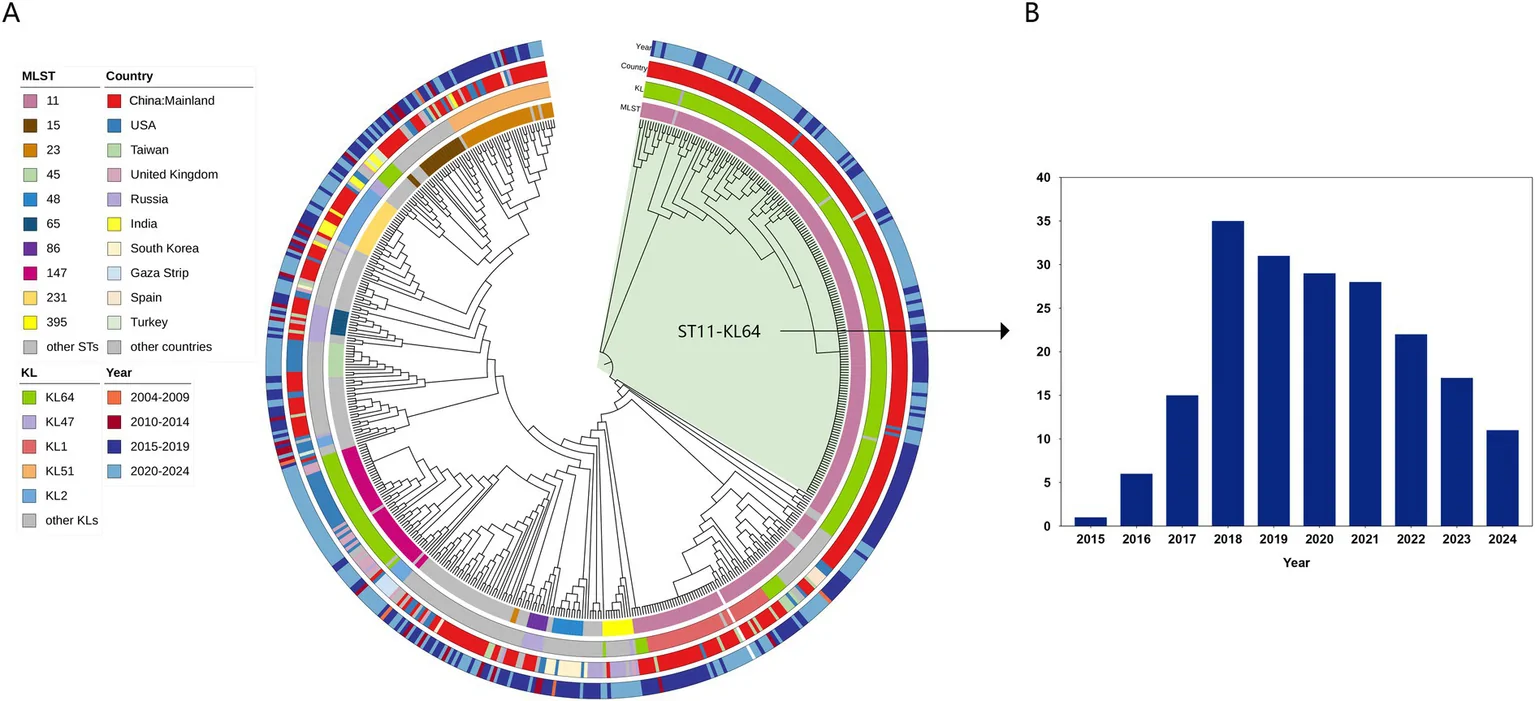
Global prevalence and phylogenetic position of the ST11-KL64 hv-CRKP clone. **(A)** Maximum-likelihood phylogeny of global hv-CRKP strains reveals a monophyletic ST11-KL64 cluster. **(B)** Temporal dynamics of ST11-KL64 hv-CRKP isolates.

A core-genome SNP phylogeny of 557 global hv-CRKP genomes (NCBI assembly accession numbers and metadata provided in Supplementary Table S1A) showed that ST11-KL64 isolates formed a distinct, densely aggregated clade. Geographic annotation indicated that a substantial fraction of genomes in this clade was from China, alongside isolates from multiple other regions (Figure 2A). Reports increased after 2018, peaked in 2018, and then fluctuated across subsequent years (Figure 2B).

#### 3.1.3 Phylogenetic placement and genomic feature matrix of the 22 highlighted local isolates

Across all isolates, the capsule locus types were KL64 (16/22, 72.7%) and KL47 (6/22, 27.3%). Two O-antigen loci were detected, with O1/O2v1 predominating (15/22, 68.2%) and OL13 present in the remainder (7/22, 31.8%). All isolates carried *bla*KPC-2 (22/22, 100%), while *bla*OXA-1 was rare (2/22, 9.1%). Hypervirulence genes were consistently detected for *iucA and rmpA2* (22/22, 100%), in contrast to the variable distribution of *rmpA* (5/22, 22.7%) and *iroB* (1/22, 4.5%), indicating heterogeneity restricted to a small subset of accessory virulence genes.

**Figure 3:**
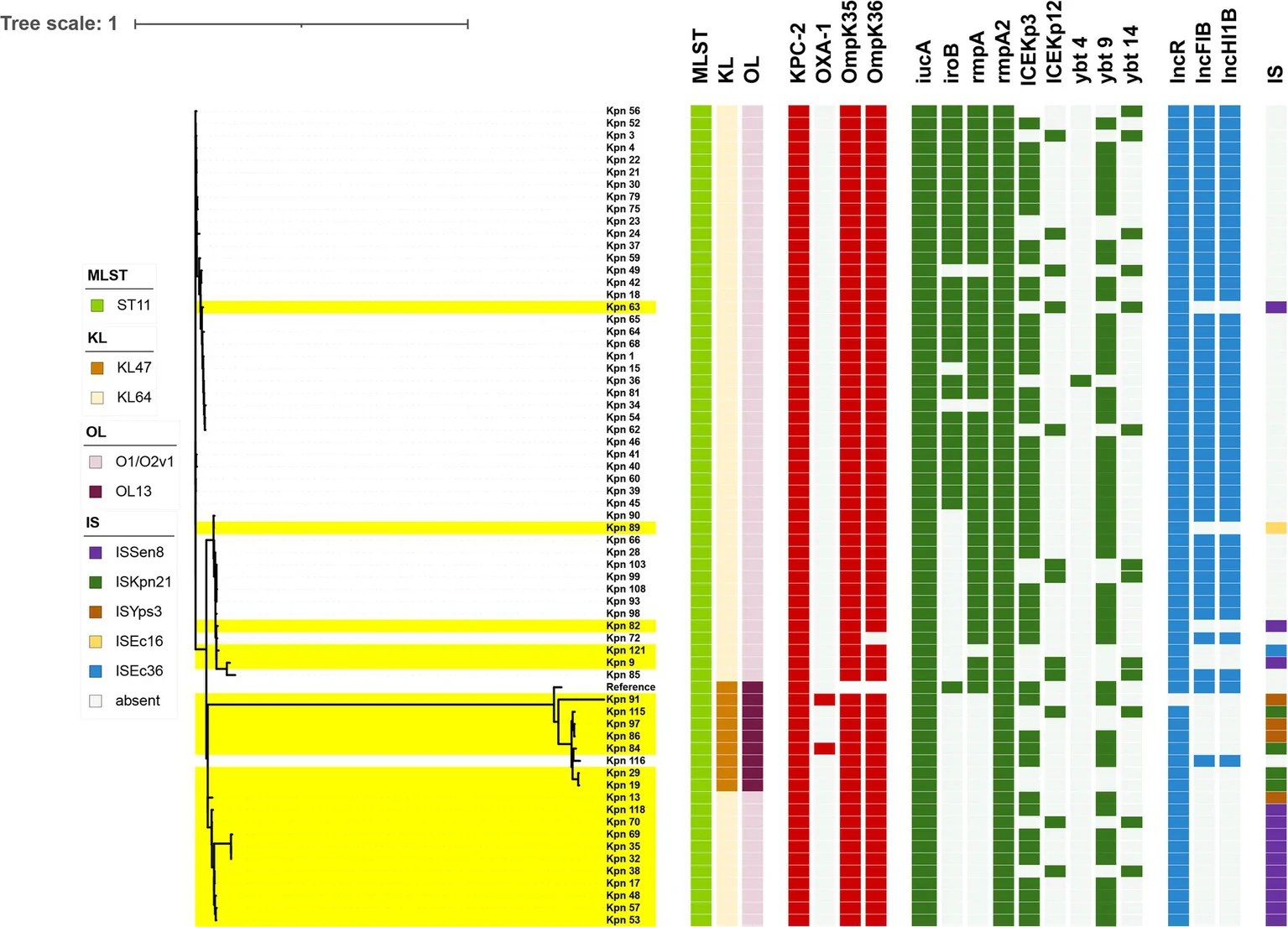
Genomic characteristics of 22 local ST11 hv-CRKP isolates with candidate chromosomal virulence integration. Yellow-highlighted isolates (*n* = 23) carried hypervirulence-associated genes but lacked the canonical virulence-plasmid replicons IncHI1B and IncFIB. Following multi-evidence validation, 22 isolates were retained as high-confidence candidates for chromosomal virulence-locus integration. Kpn_91 was excluded from the final high-confidence set because the available genomic evidence was insufficient to support its classification as a high-confidence candidate for chromosomal virulence-locus integration (Supplementary Table S4). Porin-associated features shown in the matrix were derived from Kleborate annotations: percentage-based *OmpK*35*/OmpK*36 labels indicate predicted retention of the corresponding reference protein length and should not be interpreted as confirmed nucleotide-level mutation mechanisms.

Plasmid replicon profiling further supported the shared backbone: IncR was present in all isolates (22/22, 100%), and none carried the classical virulence plasmid replicons IncHI1B or IncFIB (0/22, 0%). Several insertion sequences were observed at variable frequencies, including IS*Sen*8 (13/22, 59.1%), IS*Kpn*21 (4/22, 18.2%), IS*Yps*3 (3/22, 13.6%), and sporadic IS*Ec*16 and IS*Ec*36 (each 1/22, 4.5%). Kleborate predicted a truncated *OmpK*35 retaining approximately 17% of the reference protein length in all isolates, together with the *OmpK*36 GD loop 3 insertion in 21/22 isolates (95.5%); Kpn_89 instead showed a predicted *OmpK*36 truncation retaining approximately 72% of the reference length. These findings represent predicted porin alterations and do not specify the underlying nucleotide-level mechanism (Figure 3).

Collectively, these results indicate that the 22 local isolates shared several important genetic characteristics, including the ST11 background, universal carriage of *bla*KPC-2, *iucA* and *rmpA2*, absence of the canonical IncHI1B/IncFIB virulence-plasmid replicons, and recurrent candidate integration-associated structural features. However, variation in capsule locus, O-antigen locus, insertion-sequence content, porin alleles, and core-SNP distances indicated that the isolates were not genetically uniform. To quantify their phylogenetic relatedness, pairwise distances were calculated from a core-SNP alignment comprising 2,417 variable sites. Across the 22 isolates, pairwise SNP distances ranged from 4 to 1,756 SNPs (mean, 824.2 SNPs). Three isolate pairs differed by fewer than 15 SNPs: Kpn_17/Kpn_38 (4 SNPs), Kpn_86/Kpn_97 (8 SNPs), and Kpn_19/Kpn_29 (12 SNPs). These findings identify several closely related isolate pairs within the collection but do not support interpretation of all 22 isolates as a single recent near-clonal transmission cluster (Supplementary Tables S6A,B).

### 3.2 Breakpoint clustering and IS-associated structural variation in a candidate chromosomal virulence-associated region

**Figure 4:**
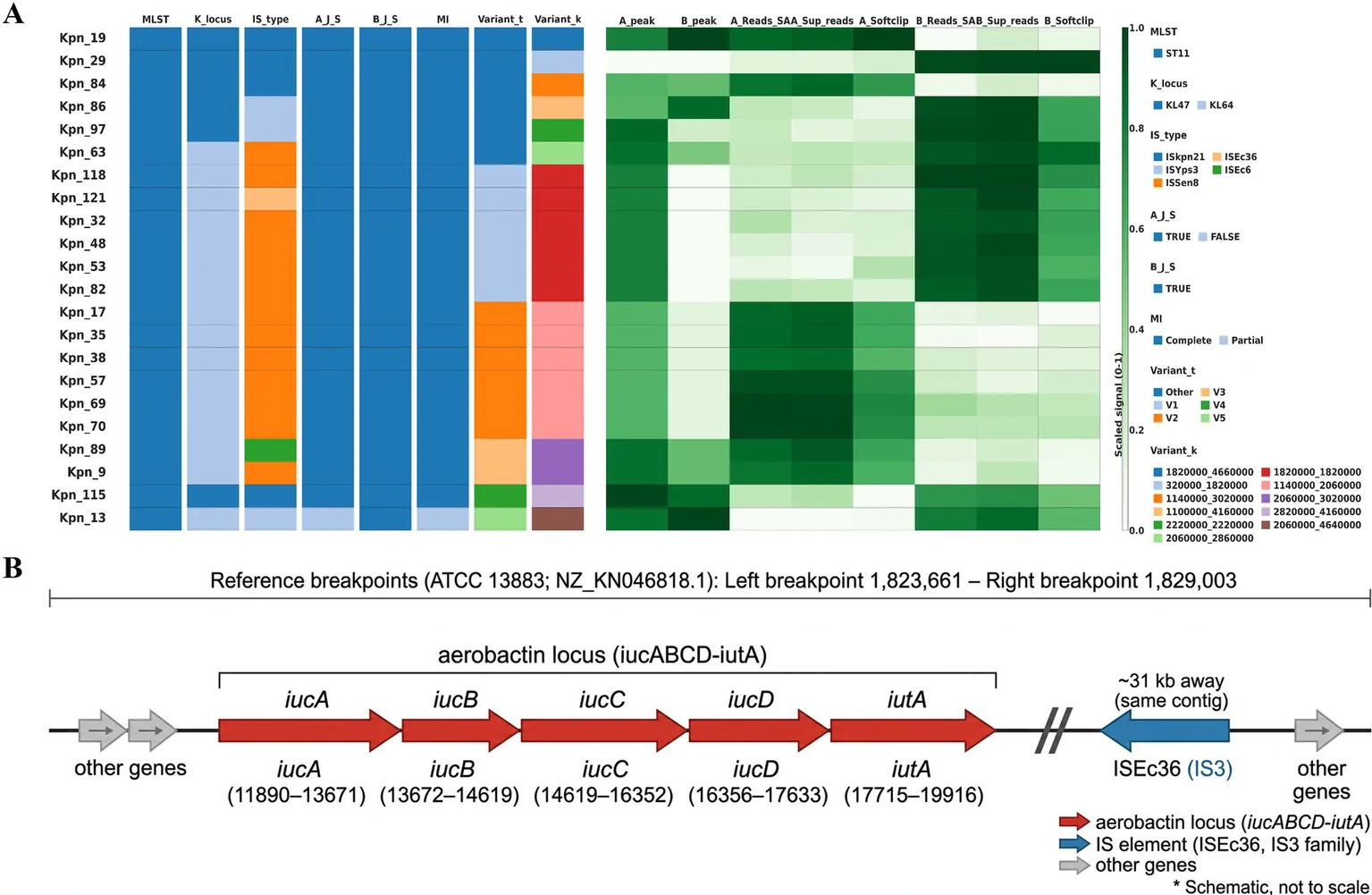
Multi-layer genomic evidence supporting chromosomal localization of virulence loci in ST11 isolates. **(A)** Heatmap summarizing genotypes and read-level evidence supporting candidate IS–chromosome junctions across 22 ST11 isolates. Rows represent individual isolates (e.g., Kpn_19 to Kpn_13). The left annotation columns indicate MLST (ST11), *K*-locus type (KL47 or KL64), and IS_type (IS*Kpn*21, IS*Ec*36, IS*Yps*3, IS*Ec*6, or IS*Sen*8), followed by junction-support status (AJ_S and B_J_S; TRUE/FALSE) and integration status (MI; Complete/Partial). The heatmap on the right shows scaled signals (0–1) for read-evidence metrics, including A_peak, B_peak, and counts of junction-supporting reads based on soft-clipped and supplementary-alignment evidence (see column headers). Abbreviations: A_J_S, a_junction_supported; B_J_S, b_junction_supported; MI, integration status (Complete/Partial); Variant_t, variant type; Variant_k, variant category, as shown. **(B)** Schematic representation of a representative chromosomal region (reference: ATCC 13883; NZ_KN046818.1) illustrating the aerobactin locus (*iucABCD–iutA*) and a nearby IS element (IS*Ec*36, IS3 family) on the same contig. The figure marks the reference breakpoints (left, 1,823,661 bp; right, 1,829,003 bp) and indicates the coordinates of genes within the aerobactin locus, as labeled: *iucA*, *iucB*, *iucC*, *iucD*, and *iutA*. Gene arrows are color-coded as shown in the figure: aerobactin-locus genes are shown in red, the IS element in blue, and flanking genes in gray. The schematic is not drawn to scale.

Across the 22 ST11 isolates, breakpoints within a candidate chromosomal virulence-associated region were non-randomly distributed and formed several recurrent patterns shared by multiple isolates, suggesting preferred breakpoint locations. Most isolates showed concordant read-level support at both inferred breakpoint ends. Specific breakpoint patterns were associated with particular IS elements: IS*Sen*8 was frequently observed, whereas additional IS types occurred in selected patterns; isolates with the KL47 capsular locus more frequently harbored IS*Kpn*21 or IS*Yps*3. Overall, these findings indicate recurrent structural variation in this chromosomal virulence-associated region and an association between specific breakpoint patterns and nearby IS elements; however, the available sequencing data do not establish a direct mechanistic role for IS elements in generating these rearrangements (Figure 4A).

Kpn_121 was used as a representative example to illustrate the local genomic context. In the reference genome ATCC 13883 (NZ_KN046818.1), the inferred breakpoint coordinates delimit a reference-mapped interval spanning 1,823,661–1,829,003 bp, encompassing the aerobactin locus *iucABCD-iutA*. An IS3 family element (IS*Ec36*) is located approximately 31 kb away on the same contig. The presence of this nearby IS element is compatible with a mobile-element-rich local genomic context. However, its role in generating the inferred breakpoint-associated structures cannot be determined from the available short-read sequencing data (Figure 4B).

### 3.3 Reference-sensitivity assessment using a closer ST11 reference

To evaluate whether the breakpoint-associated observations were dependent on the use of the genetically distant ATCC 13883 reference, we repeated the mapping analysis using the closer ST11 reference TVGHCRE225. Across the 22 isolates, the mean proportion of mapped reads increased from 82.7% with ATCC 13883 to 91.7% with TVGHCRE225, while the mean proportion of properly paired reads increased from 81.3 to 90.7%. All 22 isolates showed higher mapped-read and properly paired-read proportions with TVGHCRE225. Mean MAPQ values were also comparable or slightly higher with the closer reference. These findings indicate that TVGHCRE225 provides a more compatible mapping background for the ST11 isolates. Detailed per-isolate mapping statistics are provided in Supplementary Table S3A.

We next assessed whether the ATCC 13883-based breakpoint signatures were reproduced at homologous regions in the TVGHCRE225 assembly. The candidate regions remained mappable to the TVGHCRE225 plasmid contig CP023723.1 in all 22 isolates. However, no isolate showed complete reproduction of the original two-junction signature. Ten isolates (45.5%) retained weak residual soft-clipping or supplementary-alignment signals at one or more homologous regions, whereas 12 isolates (54.5%) were mapping-compatible only and showed no qualifying read-level junction evidence. Thus, the closer-reference analysis supports the compatibility and persistence of the candidate regions across reference backgrounds, but does not independently confirm nucleotide-resolution breakpoint structures. Per-isolate homologous-region coordinates, breakpoint-associated read-level signal summaries, and classification outcomes are provided in Supplementary Table S3B.

### 3.4 Read-level evidence and standardized support profiling for recurrent breakpoint events

#### 3.4.1 Read-level evidence in representative strains

**Figure 5:**
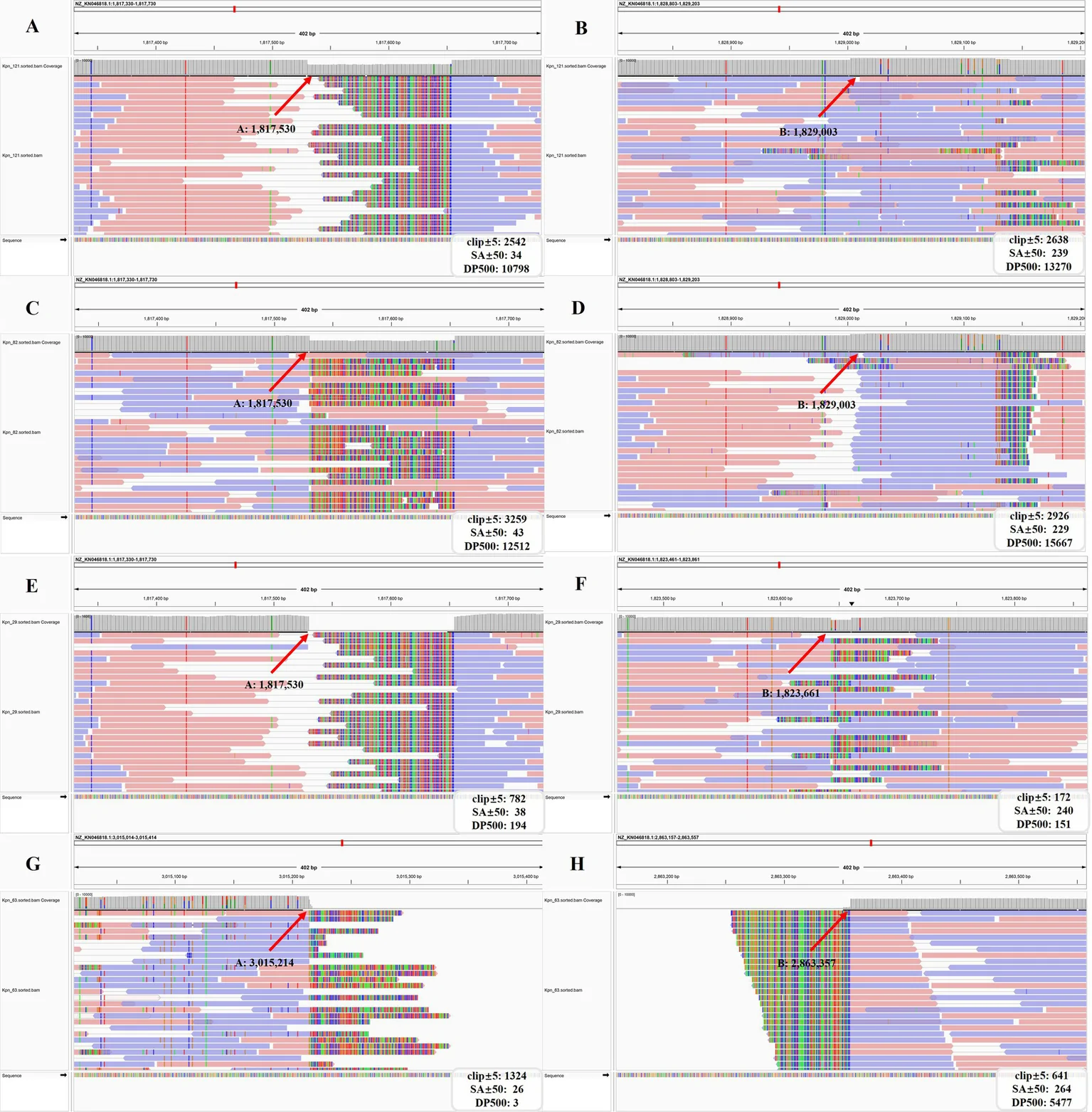
Read-level evidence for candidate integration-associated breakpoint signals in four representative ST11 strains. Eight IGV snapshots showing read alignments and coverage patterns around candidate breakpoint positions. Each panel displays an approximately 402-bp window containing the coverage track at the top, aligned reads on the forward and reverse strands in the middle (colored as shown in the IGV view), and the reference sequence at the bottom. The red arrow indicates the breakpoint coordinate. For each panel, the figure reports counts of: (i) Soft-clipped reads within ±5 bp of the breakpoint (clip±5); (ii) Supplementary-alignment reads within ±50 bp (SA ± 50), representing split-read evidence; and (iii) DP500, representing read-depth counts meeting the depth ≥500 threshold shown in the panel. Reference coordinates are shown above each IGV window. These panels are shown in the ATCC 13883 coordinate system and illustrate candidate breakpoint-associated signals rather than nucleotide-resolution validation of the corresponding junctions. **(A,B)** (Kpn_121, NZ_KN046818.1): Breakpoints at A, 1,817,530 bp, and B, 1,829,003 bp. **(C,D)** (Kpn_82, NZ_KN046818.1): Breakpoints at A, 1,817,530 bp, and B, 1,829,003 bp. **(E,F)** (Kpn_29, NZ_KN046818.1): Breakpoints at A, 1,817,530 bp, and B, 1,823,661 bp. **(G,H)** (Kpn_63, NZ_KN046818.1): Breakpoints at A, 3,015,214 bp, and B, 2,863,357 bp.

In IGV local alignments from four representative ST11-KL64 strains (covering Groups A–D), breakpoint-flanking regions showed read-level patterns compatible with structural variation, including clusters of soft-clipped reads and SA-tagged supplementary alignments near the ATCC 13883-based candidate coordinates (Figure 5). These patterns provide read-level support for local structural variation and are consistent with breakpoint-associated rearrangement signatures; however, they do not resolve the precise structure or mechanism of the inferred event. Concordantly, summary metrics such as clip±5, SA ± 50, and DP500 were increased in the same windows, providing quantitative support for the visual evidence and providing additional quantitative support for the observed reference-based signals, while not excluding reference-dependent alignment effects.

#### 3.4.2 Standardized breakpoint evidence strength and clustering patterns across 22 strains

**Figure 6:**
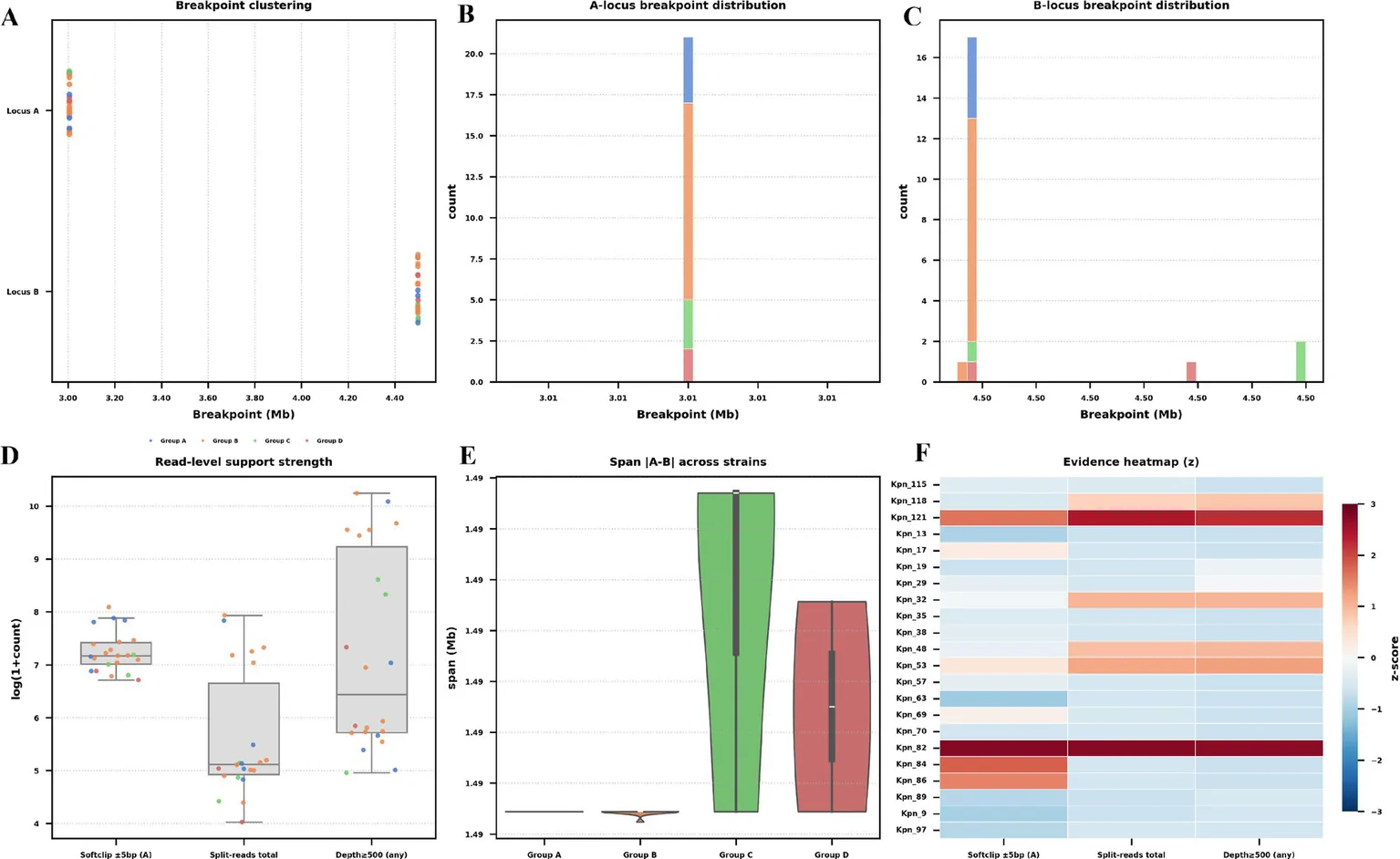
Clustering of candidate breakpoint-associated coordinates and reference-based read-level support across 22 ST11 strains. **(A)** Breakpoint-clustering scatter plot showing the genomic positions of two breakpoint loci, locus A and locus B, across strains. Points are colored by group (Groups A–D), as shown in the legend. The x-axis indicates the breakpoint position in Mb. Clustering of the points indicates that most isolates share common integration junctions. **(B)** Stacked histogram showing the distribution of breakpoint positions for locus A across strains, with counts stratified by group (Groups A–D). The x-axis indicates the breakpoint position in Mb, and the y-axis indicates the number of isolates. Different colors represent different groups, as shown in the legend. The peak indicates the most frequently observed breakpoint position at locus A among the 22 isolates. **(C)** Stacked histogram showing the distribution of breakpoint positions for locus B across strains, with counts stratified by group (Groups A–D). The x-axis indicates the breakpoint position in Mb, and the y-axis indicates the number of isolates. Different colors represent different groups, as shown in the legend. The peak indicates the most frequently observed breakpoint position at locus B among the 22 isolates. **(D)** Summary of the strength of read-level support for different types of breakpoint evidence across strains. Metrics include Softclip ±5 bp **(A)**, Split-reads total, and Depth ≥500 (any). The y-axis is shown as log(1 + count). Higher values indicate stronger support for the integration junctions. **(E)** Distribution of the genomic span between loci (|A–B|) across groups (Groups A–D). Each point represents an individual strain, and the y-axis shows the span distance in Mb. Shorter distances in certain groups suggest deletion or rearrangement of the intervening sequence between the two integration junctions. **(F)** Z-score heatmap of standardized read-evidence metrics across strains, including Softclip ±5 bp (A), Split-reads total, and Depth ≥500 (any). Rows represent strain IDs, columns represent evidence types, and color intensity indicates the relative z-score within each metric (red, higher-than-average support; blue, lower-than-average support). The heatmap allows rapid identification of strains with particularly strong or weak integration evidence.

Across all 22 strains, z-score heatmaps integrating multiple short-read evidence types around the candidate breakpoint sites and adjacent regions revealed pronounced heterogeneity in both signal strength and signal composition (Figure 6). Some isolates were characterized mainly by soft-clip enrichment, others showed predominant split-read support, and a subset exhibited concurrent increases across multiple metrics, forming a more coherent “combined breakpoint-evidence” profile. In addition, several strains displayed localized abnormally high depth signals near the breakpoint, suggesting read pile-up or coverage enrichment in these regions. Overall, breakpoint-associated signals were non-randomly distributed across strains and clustered predominantly at two recurrent genomic loci, consistent with recurrent candidate structural configurations and possible biological relatedness among isolates. We quantified breakpoint read signals and local depth at A/B sites across 22 strains, with details in Supplementary Table S7.

#### 3.4.3 Coverage assessment supports adequate read coverage across the candidate integration-associated region

**Figure 7:**
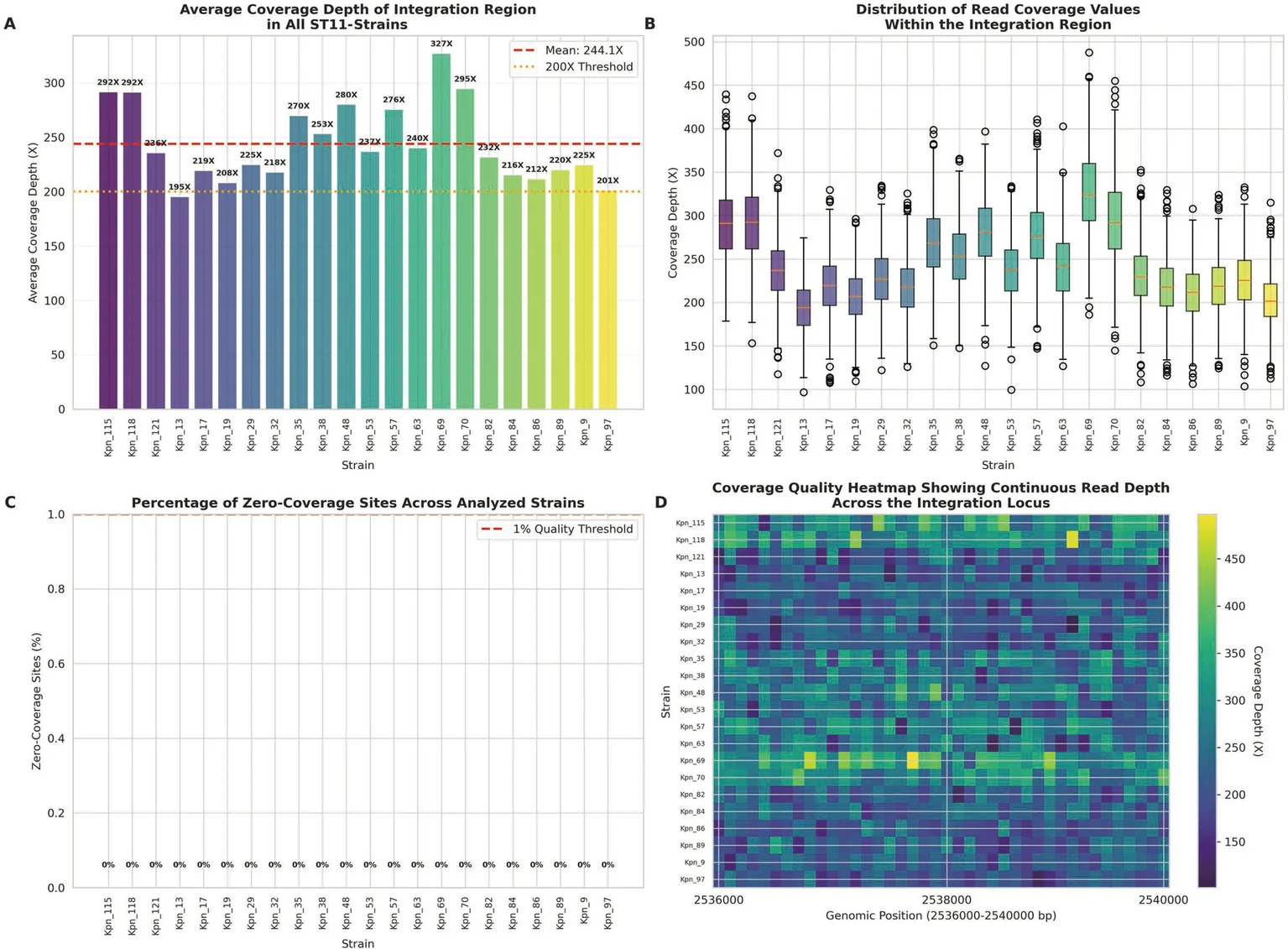
In-depth assessment of sequencing coverage across the virulence-gene integration region. **(A)** Bar plot of the average sequencing coverage depth across the integration region for all analyzed ST11 strains (22 strains, as indicated). The red dashed line indicates the overall mean coverage (mean, 244.1×), and the orange dashed line indicates the 200 × quality threshold. All strains show coverage well above the 200 × threshold, supporting the reliability of integration-junction detection. **(B)** Boxplots showing the distribution of per-site read-coverage depths within the integration region for each strain. Each box represents the interquartile range (IQR) of coverage across all positions within the integration region for that strain, and the horizontal line within each box indicates the median coverage. **(C)** Percentage of zero-coverage sites within the integration region across strains. The dashed line indicates the 1% quality threshold. All strains showed ≤1% zero-coverage sites, confirming that the integration region was reliably covered by sequencing reads. **(D)** Coverage heatmap showing continuous read depth across the integration locus, with strains on the y-axis and genomic positions on the x-axis (displayed window, 2,536,000–2,540,000 bp). The color scale represents coverage depth (×), demonstrating the continuity of sequencing support across the locus. Yellow indicates high coverage, whereas blue indicates low coverage. The continuous yellow blocks across rows indicate stable, high-quality coverage across the integration region.

Coverage-completeness analyses further support the continuity and reliability of short-read data across the target integration region (Figure 7). Sequencing depth in this region was generally high (mean approximately 244×, with most strains >200×), and the proportion of zero-coverage positions was 0% in all strains, indicating no zero-coverage positions within the analyzed reference-mapped interval. The coverage heatmap showed smooth, continuous depth profiles along the region, suggesting the absence of systematic blind spots due to low coverage or missing data near key breakpoints and the candidate integration region. Together, these results provide a stable sequencing basis for subsequent breakpoint inference related to structural variation, and the strain-level background can be cross-referenced with the corresponding summaries in the supplementary Table S4.

### 3.5 Sequence comparison with reference virulence plasmids

To further characterize the candidate chromosome-associated virulence region, we performed BLASTn alignment of the extracted *iucABCD-iutA* region from all 22 ST11 isolates against the canonical virulence plasmids pLVPK (AY378100) and pK2044 (NC_006625). All isolates showed ≥99.9% nucleotide identity to pK2044 over extended regions (~24–77 kb); most isolates showed ≥99.8% identity to pLVPK (~24–68 kb), with one exception (Kpn_13, 99.425% identity to pLVPK despite a long best-hit alignment), consistent with natural sequence divergence among reference virulence plasmids. In contrast to the plasmid-borne configuration, where the aerobactin locus is flanked by plasmid backbone genes (repA, parA, and tra-associated genes), the candidate chromosome-associated virulence-locus configurations in all 22 isolates showed flanking or nearby IS elements (ISEc36, ISKpn21, ISYps3, and ISSen8), with no plasmid backbone genes or replicons (IncFIB/IncHI1B) detected in the flanking regions. These results show that the candidate chromosome-associated *iucABCD-iutA* region is highly conserved and homologous to plasmid-borne aerobactin loci. The data are summarized in Supplementary Tables S8A,B and illustrated in Supplementary Figure S2.

### 3.6 Virulence phenotypes and evolutionary pathways of hv-CRKP

#### 3.6.1 Virulence in the *Galleria mellonella* infection model

**Figure 8:**
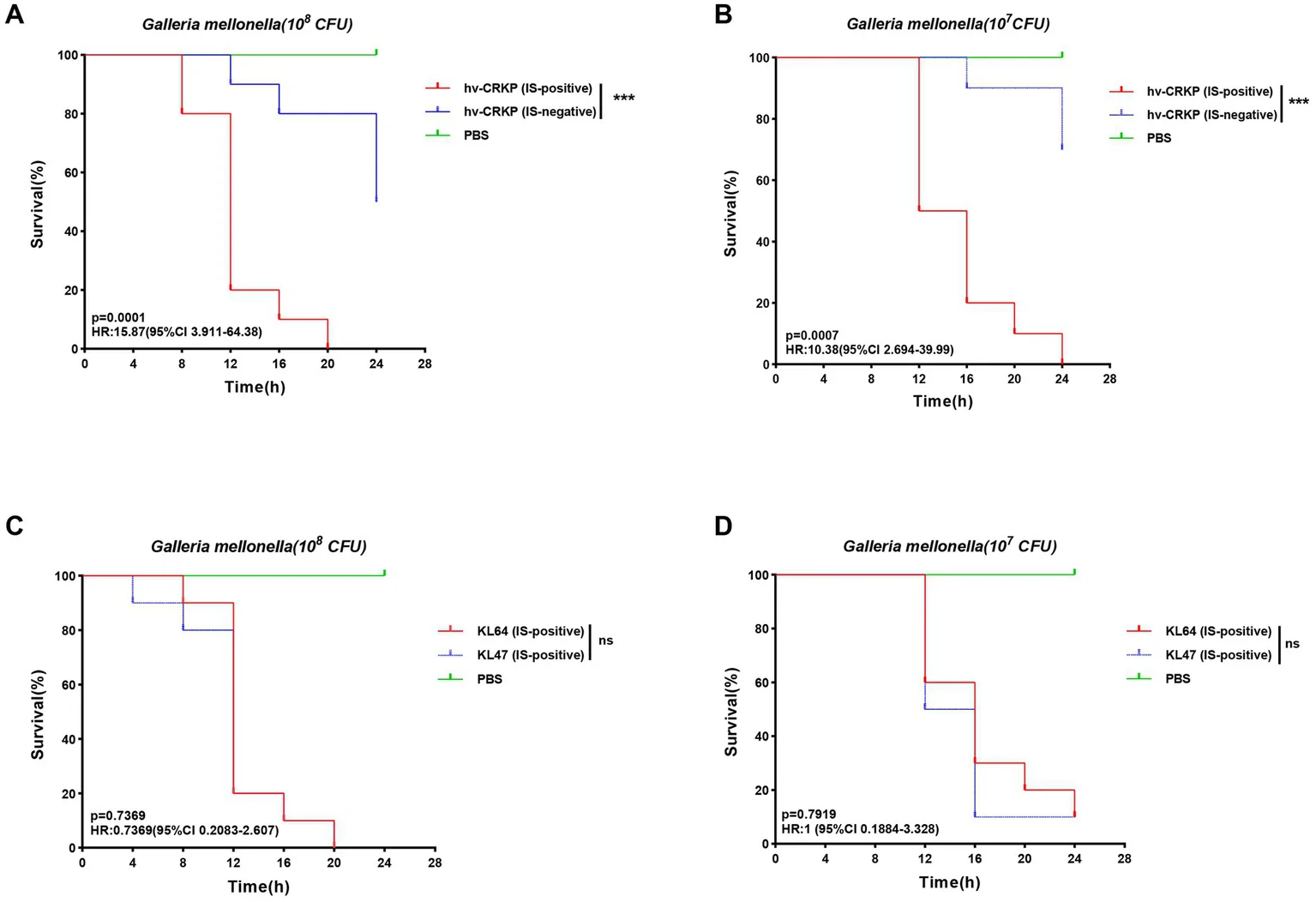
Virulence assessment of hv-CRKP strains in a *Galleria mellonella* infection model. **(A)** Survival curves of larvae infected with high doses (10^6^ CFU/larva) of hv-CRKP (IS-positive) versus hv-CRKP (IS-negative) strains. **(B)** Survival curves of larvae infected with low doses (10^5^ CFU/larva) of hv-CRKP (IS-positive) versus hv-CRKP (IS-negative) strains. **(C)** Survival curves of larvae infected with high doses of KL64 (IS-positive) versus KL47 (IS-positive) strains. **(D)** Survival curves of larvae infected with low doses of KL64 (IS-positive) versus KL47 (IS-positive) strains. The IS-positive hv-CRKP group comprised 15 distinct strains, including 8 KL64 and 7 KL47 strains, whereas the IS-negative hv-CRKP group comprised 10 distinct strains. Each strain was tested using 10 larvae per replicate in three independent biological replicates (30 larvae per strain in total). PBS-injected larvae served as the non-infected control. *p*-values were calculated using the log-rank (Mantel–Cox) test and are shown in each panel; hazard ratios (HRs) and 95% confidence intervals (CIs) are indicated in the corresponding panels.

We used a *Galleria mellonella* infection model to compare virulence phenotypes among hv-CRKP strains with different IS-associated classifications (Figure 8). The IS-positive and IS-negative comparator groups comprised 15 and 10 distinct strains, respectively; the IS-positive group included 7 IS-positive KL47 strains and 8 IS-positive KL64 strains. At both inoculum doses (10^8^ and 10^7^ CFU), IS-positive hv-CRKP isolates caused significantly faster and higher mortality than those of IS-negative hv-CRKP isolates and the PBS control. At 10^8^ CFU, survival in the IS-positive group fell to 0% by 20 h (*p* = 0.0001; HR = 15.87, 95% CI: 3.911–64.38; Figure 8a). Likewise, at 10^7^ CFU, survival reached 0% by 24 h (*p* = 0.0007; HR = 10.38, 95% CI: 2.694–39.99; Figure 8b). When further comparing the two IS-positive isolates KL64 and KL47, both showed time-dependent mortality but did not differ significantly at either dose (10^8^ CFU: *p* = 0.7369; Figure 8c; 10^7^ CFU: *p* = 0.7919; Figure 8d). These results indicate no statistically significant difference in survival between the selected KL64 and KL47 isolates in this pairwise comparison.

#### 3.6.2 Comparison of classical fusion-plasmid architectures and candidate chromosome-associated virulence-locus configurations in hv-CRKP

**Figure 9:**
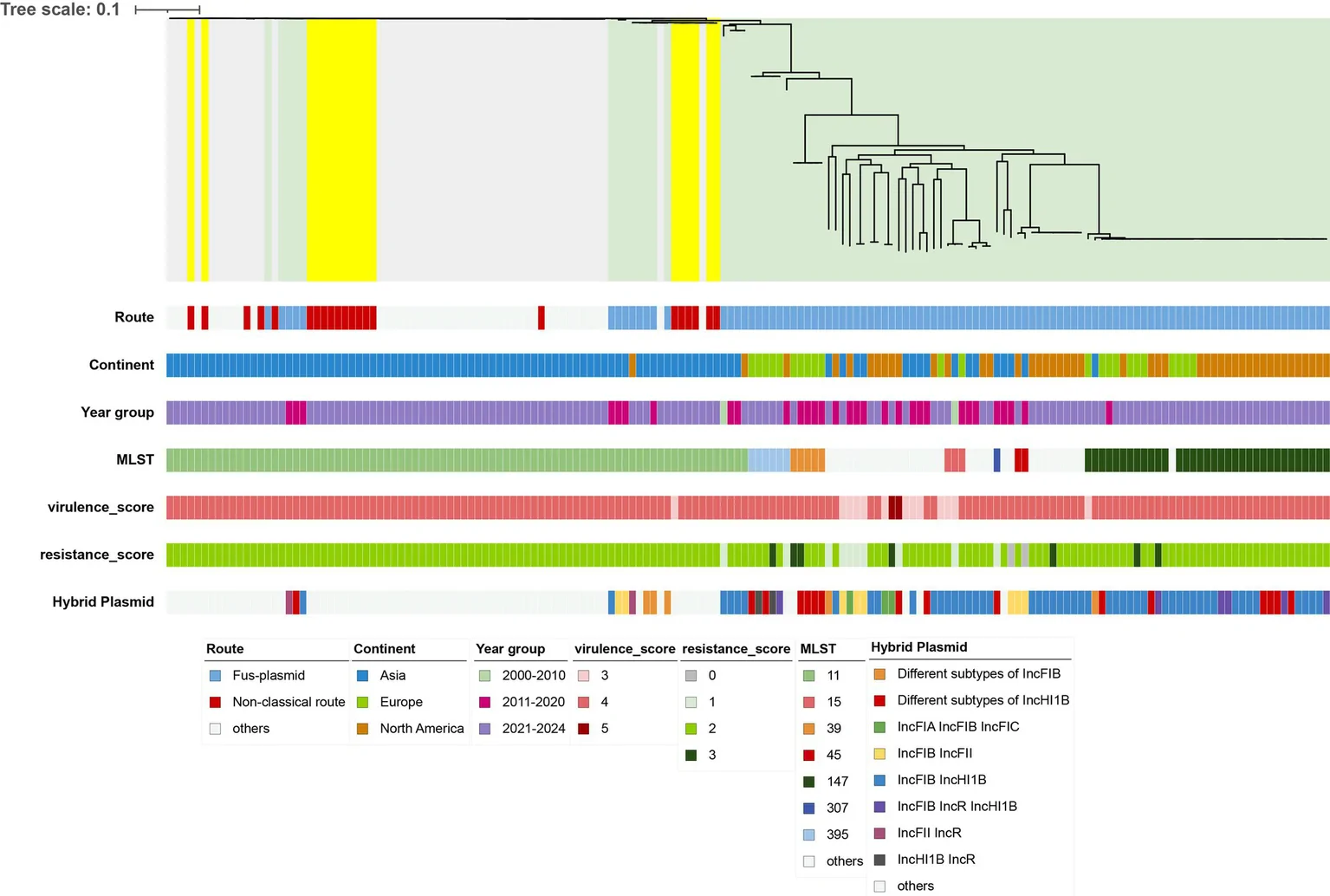
Core-genome phylogeny of genetic backgrounds and hybrid plasmid profiles among hv-CRKP.

Within the analyzed global dataset, hv-CRKP genomes showed two broad genomic patterns: isolates with canonical fusion-plasmid features and isolates carrying candidate chromosome-associated virulence-locus configurations (Figure 9). In the currently available dataset, genomes with canonical fusion-plasmid features were more often represented by isolates reported from Europe and North America, whereas genomes carrying candidate chromosome-associated configurations were predominantly represented by isolates from China and were more frequently sampled after 2021. These geographic and temporal patterns may partly reflect variation in genomic surveillance and public-database sampling. The former pattern occurred across multiple sequence types, such as ST147, ST307, and ST395. The latter pattern mainly involved strains from China, mostly collected after 2021, and was dominated by ST11. Their Kleborate-derived genomic virulence and resistance scores were highly consistent (virulence score = 4; resistance score = 2). Typical IncFIB/IncHI1B replicon signals were not detected in their virulence-associated sequences.

Mash-based plasmid phylogenetic analysis further supports this distinction (Supplementary Figure S1). GenBank accession numbers for reference fusion plasmids and *bla*KPC-2-carrying plasmids from this study are provided in Supplementary Tables S1B,C, respectively. The *bla*KPC-2 plasmids from the 22 strains in this study form a single cluster and are separated from the global classical fusion plasmids, suggesting distinct evolutionary relationships. Together, these results support a distinction between canonical fusion-plasmid architectures and the candidate chromosome-associated virulence-locus configurations identified in this study with respect to ST background, the available geographic and temporal metadata, virulence-plasmid replicon profiles, and *bla*KPC-2-plasmid relatedness.

#### 3.6.3 Conceptual model: canonical fusion-plasmid architecture and a candidate chromosome-associated virulence-locus configuration

**Figure 10:**
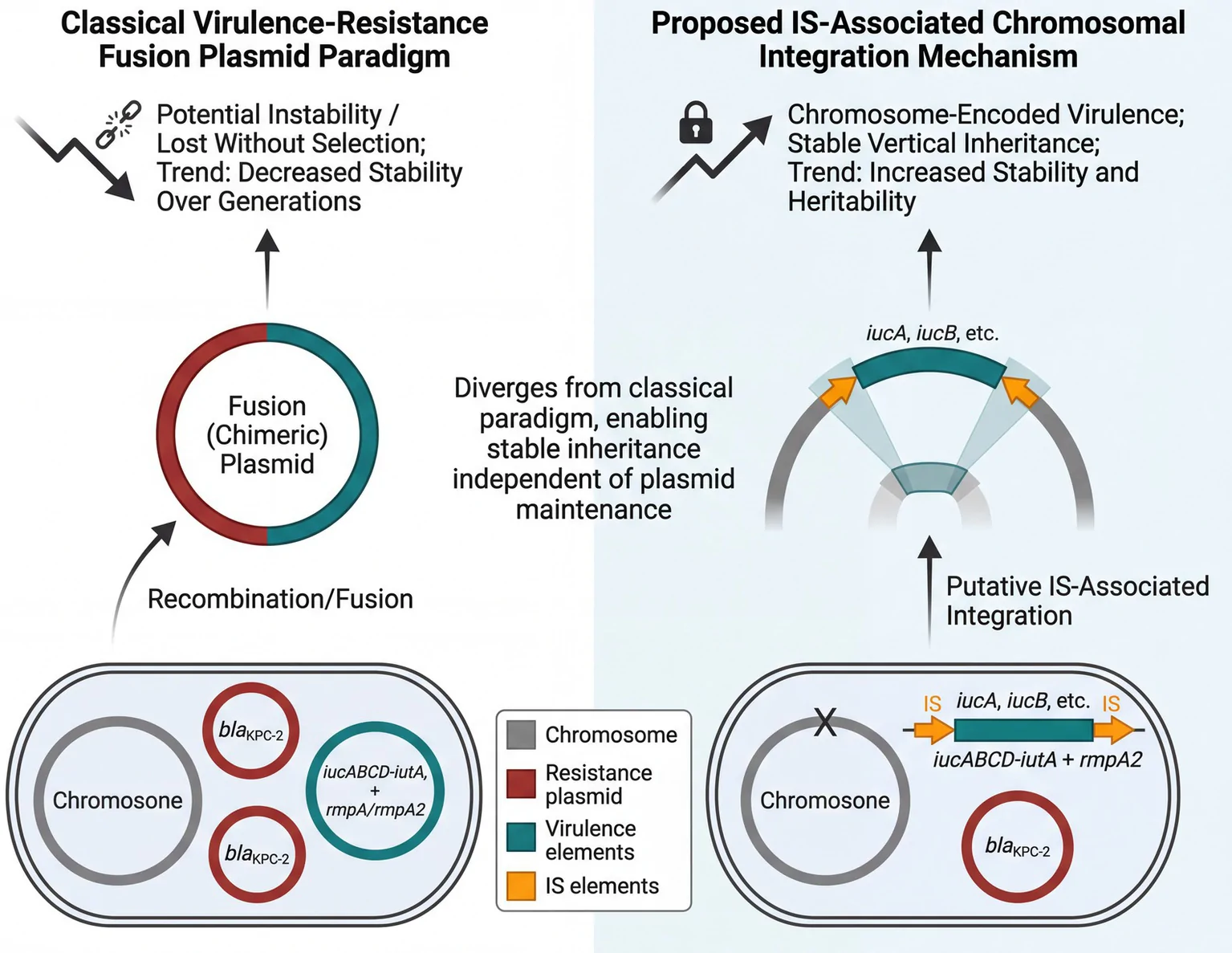
Schematic model of two evolutionary pathways leading to hv-CRKP.

Based on short-read sequencing data, phenotypic observations, and phylogenetic classification, we outline two putative genomic configurations that may be associated with genomic convergence of genetically defined hv-CRKP isolates (Figure 10).

In the previously described fusion-plasmid model, a resistance plasmid carrying *bla*KPC-2 and a virulence plasmid carrying *iucABCD–iutA* together with *rmpA/rmpA2* may recombine to form a hybrid plasmid. Although hybrid plasmids have been reported to undergo structural changes or loss under certain conditions, their stability in the isolates analyzed here was not directly assessed (Figure 10, left).

In an alternative candidate chromosome-associated virulence-locus configuration, virulence-associated loci (e.g., *iucABCD–iutA* and *rmpA2*) may be chromosomally located in genomic regions containing nearby IS elements. This configuration could reduce dependence on maintenance of a canonical virulence plasmid; however, the available short-read sequencing data do not establish the precise integration mechanism, the direct role of IS elements, or the stability of the inferred chromosomal configurations (Figure 10, right).

## 4 Discussion

Our study builds on previous reports of chromosomal localization of hypervirulence-associated determinants, including the aerobactin-associated *iucABCD–iutA* locus, in *K. pneumoniae* (Wei et al., 2025; Yang et al., 2020). By integrating genomic localization analyses, comparative genomics, and phenotypic assays, we provide convergent sequencing-based evidence consistent with putative IS-associated chromosomal localization of virulence gene clusters in the ST11-KL64 hv-CRKP lineage examined here. Notably, high-risk hv-CRKP phenotypes within ST11 are not confined to KL64; dissemination of ST11-O13/KL47 hypervirulent carbapenem-resistant isolates has also been reported, underscoring ongoing diversification of capsular backgrounds within the ST11 epidemic pool (Liu et al., 2025). Importantly, the integrated region was not uniform across strains; instead, multiple recurrent integration architectures (Patterns A–D) were observed, suggesting ongoing structural diversification rather than a single idiosyncratic event. This observation is consistent with the heterogeneous horizontal gene-transfer dynamics and extensive accessory-genome plasticity described across drug-resistant and virulent *K. pneumoniae* lineages (Zhang, et al., 2020; Zhou et al., 2020; Wyres et al., 2019).

A key strength of this work is the multi-criterion support for chromosomal localization despite the inherent limitations of short-read sequencing. Across the 22 supported strains, the inferred integration regions were assembled in long contigs spanning the locus of interest, showed continuous read coverage under our mapping criteria, and exhibited read-level signatures consistent with chromosomal junctions, including split/supplementary and soft-clipped alignments at the inferred breakpoints. The contigs contained chromosomal-context genes, including core housekeeping genes, and did not show assembly-graph features consistent with canonical circular virulence plasmids. Together with the recurrence of shared breakpoint patterns across multiple isolates, these observations are consistent with the possibility that the virulence clusters are chromosome-associated rather than exclusively plasmid-borne (Wyres et al., 2020; Lam et al., 2021).

These findings extend the classical model of hv-CRKP evolution, which emphasizes the emergence of hypervirulent, carbapenem-resistant strains through acquisition of resistance or virulence plasmids and, in some cases, the formation of resistance–virulence hybrid plasmids (Yang et al., 2021; Tian et al., 2021; Han et al., 2022; Navon-Venezia et al., 2017; Lan et al., 2021). Such convergence is not restricted to ST11 or to China; a single plasmid vector carrying both virulence- and multidrug-resistance-associated determinants has also been described in MDR *K. pneumoniae* ST15, illustrating the diversity of plasmid-mediated evolutionary routes (Lam et al., 2019). Notably, acquisition of conjugative MDR–hypervirulent plasmids can directly confer hypervirulence in clinical CRKP (Li et al., 2023). Our data support an additional, non-exclusive putative evolutionary model in which virulence loci are chromosomally located while resistance determinants remain plasmid-borne, consistent with reports from China of both convergent plasmids and chromosomal integration (Li et al., 2025). Such a division of labor between the chromosome and plasmids could generate high-risk genotypes through routes other than large chimeric plasmids and may contribute to the persistence and dissemination of certain lineages.

Within our dataset, an important clue supporting an alternative genetic context for virulence loci was the consistent detection of hypervirulence-associated gene content and corresponding virulence-related phenotypes in strains lacking canonical pLVPK-like virulence-plasmid signatures. This observation prompted us to investigate whether the relevant virulence loci were located outside a canonical virulence plasmid backbone (Gu et al., 2018; Russo and Marr, 2019). More broadly, isolates carrying both MDR- and hypervirulence-associated genetic features can nevertheless exhibit unexpectedly low virulence, cautioning against inferring virulence from gene markers alone (Kochan et al., 2023; Harada and Doi, 2018; Choby et al., 2020). Accordingly, the chromosomal location of a virulence locus should not itself be interpreted as proof of hypervirulence, because the relevant phenotype may also depend on locus integrity, regulatory context, gene expression, and the broader genomic background. This pattern is not fully explained by a strictly plasmid-dependent model and is consistent with chromosomal localization or capture of virulence loci. Consistent with this observation, phylogenetic analyses placed isolates carrying the candidate integration structures within a related ST11-associated genetic background that was distinguishable from other circulating ST11 clades in which hybrid plasmids have been described (Zhang, et al., 2020; Liu et al., 2022). However, the pairwise core-SNP analysis also revealed substantial diversity within the 22-isolate collection, indicating that these isolates should not be interpreted as a single recent near-clonal transmission cluster. Collectively, these results highlight that convergent Hv-CRKP phenotypes can arise through genetically distinct routes.

The observed heterogeneity of integration structures, including the presence of multiple recurrent patterns and the predominance of one pattern in our collection, raises the possibility that certain chromosomal configurations may be more stable or less costly than others. However, frequency alone does not prove a selective advantage. The fitness consequences, stability, and transmission impact of each integration pattern should be tested directly, for example by measuring the stability of virulence loci under non-selective conditions, growth and competition *in vitro*, and virulence and transmission-associated traits *in vivo*. Such experiments will be essential to distinguish between neutral drift, founder effects, and true adaptive benefits.

Clinically, our findings argue that surveillance frameworks focusing only on plasmid replicons or known virulence plasmid backbones may miss clinically relevant chromosomal virulence configurations (Lam et al., 2021; Kochan et al., 2023). A more complete genomic surveillance strategy should incorporate virulence-locus sequence typing, markers of IS-associated virulence loci, and their chromosomal neighborhoods, enabling detection of high-risk strains even when canonical virulence plasmids are absent. Such an approach complements existing genome-based surveillance frameworks designed to track lineage, including Kleborate- and PathogenWatch-based approaches, resistance, virulence loci, and mobile genetic elements across diverse *K. pneumoniae* populations (Argimón, et al., 2021; Lam et al., 2021). From an infection-control perspective, chromosomal encoding of virulence determinants may increase the persistence of hypervirulence traits by reducing reliance on plasmid maintenance, potentially complicating efforts to curb dissemination once such lineages become established. Separately, variation in virulence plasmids has been linked to cross-regional transmission of ST11-KL64 hv-CRKP (Hong et al., 2024). In addition, within-host treatment pressure may drive resistance evolution in ST11 hv-CRKP, further complicating clinical management (Jin et al., 2021).

The structural comparison with reference virulence plasmids further reinforces the distinct nature of the integration events in our study. The *iucABCD-iutA* locus itself is highly conserved between the chromosomal and plasmid-borne copies (≥99.8% identity), but the flanking context differs fundamentally—plasmid backbone genes versus IS elements. This architecture, combined with the absence of canonical virulence plasmid replicons, argues against the simple integration of an intact canonical virulence plasmid and is consistent with a putative IS-associated capture or rearrangement involving the virulence gene cluster. The recurrence of this configuration across 22 ST11 isolates, including both KL64 and KL47 capsule types, suggests that related candidate IS-associated chromosomal configurations recur in this collection. Their stability and evolutionary consequences require direct experimental testing.

A reference-related limitation also warrants consideration. The initial breakpoint-associated analysis used ATCC 13883, which is not an ST11 reference and may introduce lineage-dependent mapping effects. To address this concern, we repeated the analysis using the closer ST11 reference TVGHCRE225. The higher mapped-read and properly paired-read proportions obtained with TVGHCRE225 indicate improved reference compatibility. Nevertheless, the closer-reference analysis yielded no complete reproduction of the original junction signatures; 10 of 22 isolates retained only weak residual signals, and 12 of 22 were mapping-compatible without qualifying junction evidence. Accordingly, the ATCC 13883-based read patterns should be interpreted as candidate breakpoint-associated evidence rather than definitive proof of conserved breakpoint structures across reference backgrounds. This result further emphasizes the need for long-read sequencing or orthogonal junction validation.

This study has several limitations. Because most genomes were derived from short-read sequencing, repeated elements and complex structural variation may remain incompletely resolved, and the precise integration boundaries and local rearrangements cannot be fully defined. Long-read sequencing will be required to reconstruct complete chromosomal contexts, confirm integration coordinates at nucleotide resolution, and evaluate possible effects on neighboring genes. Furthermore, although our inference is supported by multiple sequencing-based criteria, including breakpoint recurrence across isolates, read-level junction signatures, and continuous coverage across the inferred integration regions, junction-flanking PCR was not performed to provide orthogonal experimental validation of the inferred insertion events. To facilitate future experimental assessment, we designed *in silico* PCR primer pairs targeting genomic regions surrounding the inferred integration sites at loci A and B; the primer sequences, predicted amplicon sizes, and *in silico* specificity assessments are provided in Supplementary Table S9. Finally, the molecular mechanisms underlying IS association and the contribution of chromosomal integration to fitness and virulence remain to be experimentally validated.

In conclusion, our study expands a plasmid fusion–centric view of hv-CRKP evolution by providing convergent sequencing-based evidence for putative IS-associated chromosomal localization of virulence determinants in the examined ST11 lineage. Recognizing that high-risk phenotypes can emerge through both plasmid- and chromosome-centered mechanisms has direct implications for genomic surveillance, mechanistic research, and infection control strategies.

## Data Availability

The datasets presented in this study can be found in online repositories. The SRA, BioSample, and GenBank accession numbers for each strain are provided in Supplementary Table S10.

## Supplementary material

SUPPLEMENTARY FIGURE S1 Mash-based phylogenetic tree of blaKPC-2-carrying resistance plasmids from this study and reference fusion plasmids.

SUPPLEMENTARY FIGURE S2 Structural comparison of the chromosomally integrated iucABCD-iutA locus versus reference virulence plasmids pLVPK and pK2044. Gene organization of the iucABCD-iutA region in pLVPK (AY378100), pK2044 (NC_006625), and four representative ST11 isolates (Kpn_121, Kpn_82, Kpn_29, and Kpn_63) covering Groups A–D. The iucABCD-iutA gene cluster is shown in gray; plasmid backbone genes (repA, parA) are shown in red; IS elements are shown in blue. The core virulence genes are highly conserved (>99.8% identity to both pLVPK and pK2044), whereas the flanking regions differ markedly, plasmid backbone genes vs. IS elements, supporting a putative IS-associated chromosome-linked configuration. Gene arrow lengths are schematic and not drawn to scale.

## Glossary

A_J_S: A_junction_supported
ABRicate: A software tool for rapid mass screening of antimicrobial resistance and virulence genes in bacterial genomes
ABySS: Assembly by Short Sequences (genome assembly software)
B_J_S: B_junction_supported
*bla*KPC/*bla*NDM/*bla*OXA/*bla*IMP/*bla*VIM: Beta-lactamase resistance genes
bp: Base pair
BWA-MEM: Burrows-Wheeler Aligner Maximal Exact Match
CFU: Colony-forming unit
CI: Confidence interval
CISA: Contig Integration Scanner Assembly
CLSI: Clinical and Laboratory Standards Institute
CR: Carbapenem-resistant
CRKP: Carbapenem-resistant *Klebsiella pneumoniae*
DNA: DEOXYRIBONUCLEIC acid
DP500: Local read-depth calculated over a 500-bp window
GD: Gly-Asp (referring to the *OmpK36* loop 3 insertion)
HR: Hazard ratio
hv: Hypervirulent
hv-CRKP: Hypervirulent carbapenem-resistant *Klebsiella pneumoniae*
hvKP: Hypervirulent *Klebsiella pneumoniae*
ICEKp: Integrative and conjugative element in *K. pneumoniae*
IGV: Integrative Genomics Viewer
IncFIB/IncHI1B/IncR: Incompatibility plasmid replicon types
IQR: Interquartile range
IS: Insertion sequence
*iuc*/*iro*: Aerobactin and salmochelin loci, respectively
kb: Kilobase pair
KL: K-locus (capsule locus)
MALDI-TOF MS: Matrix-assisted laser desorption/ionization time-of-flight mass spectrometry
MAPQ: Mapping quality
MDR: Multidrug-resistant
MGE: Mobile genetic element
MI: Integration status (Complete/Partial)
MIC: Minimum inhibitory concentration
mlplasmids: A tool to predict plasmid- and chromosome-derived sequences
NCBI: National Center for Biotechnology Information
OL: O-locus (O-antigen locus)
*ompK35*/*ompK36*: Outer membrane porin K35/K36 genes
PBS: Phosphate-buffered saline
PCR: Polymerase chain reaction
qPCR: Quantitative polymerase chain reaction
SA: Supplementary alignment
SDS: Sodium dodecyl sulfate
SNP: Single nucleotide polymorphism
SOAPdenovo/SPAdes: Genome assembly software
ST: Sequence type
tBLASTn: Translated Basic Local Alignment Search Tool against nucleotide database
*tra*: Transfer-associated genes
Variant_k: Variant category
Variant_t: Variant type
VFDB: Virulence Factor Database
WGS: Whole-genome sequencing
